# Comprehensive benchmarking of SNV callers for highly admixed tumor data

**DOI:** 10.1371/journal.pone.0186175

**Published:** 2017-10-11

**Authors:** Regina Bohnert, Sonia Vivas, Gunther Jansen

**Affiliations:** Molecular Health GmbH, Heidelberg, Germany; CNR, ITALY

## Abstract

Precision medicine attempts to individualize cancer therapy by matching tumor-specific genetic changes with effective targeted therapies. A crucial first step in this process is the reliable identification of cancer-relevant variants, which is considerably complicated by the impurity and heterogeneity of clinical tumor samples. We compared the impact of admixture of non-cancerous cells and low somatic allele frequencies on the sensitivity and precision of 19 state-of-the-art SNV callers. We studied both whole exome and targeted gene panel data and up to 13 distinct parameter configurations for each tool. We found vast differences among callers. Based on our comprehensive analyses we recommend joint tumor-normal calling with MuTect, EBCall or Strelka for whole exome somatic variant calling, and HaplotypeCaller or FreeBayes for whole exome germline calling. For targeted gene panel data on a single tumor sample, LoFreqStar performed best. We further found that tumor impurity and admixture had a negative impact on precision, and in particular, sensitivity in whole exome experiments. At admixture levels of 60% to 90% sometimes seen in pathological biopsies, sensitivity dropped significantly, even when variants were originally present in the tumor at 100% allele frequency. Sensitivity to low-frequency SNVs improved with targeted panel data, but whole exome data allowed more efficient identification of germline variants. Effective somatic variant calling requires high-quality pathological samples with minimal admixture, a consciously selected sequencing strategy, and the appropriate variant calling tool with settings optimized for the chosen type of data.

## Introduction

The promise of personalized cancer medicine is to formulate effective treatment options based on the individual genetic makeup of patient and tumor [1–5]. Understanding the genetic mechanisms underlying tumor etiology and matching these with actionable drugs, however, remains a considerable challenge [6, 7]. Take the example of cetuximab, a monoclonal antibody that curbs proliferative signaling of the epithelial growth factor receptor EGFR. In a 2007 study, cetuximab provided a marginal but statistically significant improvement over existing chemotherapies for patients carrying EGFR mutations [8]. A subsequent study [9] revealed that about 40% of patients with EGFR mutations carry additional mutations in the KRAS gene downstream from EGFR, which preclude any benefits from cetuximab therapy. Failure to consider all cancer-relevant mutations may thus severely undermine the effectiveness of personalized treatment recommendations.

This issue has immediate consequences for the methodologies used to analyze next-generation sequencing data from tumors. They must be able to reliably separate a few tens to hundreds of tumor-relevant mutations from thousands of mostly non-informative germline mutations [10, 11], and must do so despite the complex and distinct biology of individual tumors and despite the technical issues associated with large next generation sequencing data sets. Separation of germline and somatic mutations is especially hampered by distinct allele frequency distributions in tumor and germline. Many cancer-relevant variants are present at low frequencies due to varying degrees of genetic heterogeneity in the tumor [12–14], i.e., the accumulation of novel mutations as cancer cells continue to divide within the growing tumor. Moreover, typical pathological tumor samples often include a considerable proportion of non-cancerous stromal and immune cells that decrease the overall frequency of somatic mutations. Consequently, frequencies of cancer variants in tumor samples may drop so low that they may be virtually indistinguishable from sequencing artefacts. It is therefore pivotal to optimize the variant calling step at the start of any personalized medicine pipeline.

Ideally, the pipeline identifies all cancer-relevant mutations present in the tumor (maximal sensitivity) but avoids calling misleading false positives due to artifacts and contaminations in the raw data (maximal precision). Previous comparisons of SNV callers [15–20] already highlighted that available calling methods often yield highly discordant results. However, these studies did not systematically compare somatic and germline SNV callers on whole exome and targeted gene panel data, especially taking into account distinct parameter settings for each tool. Moreover, the influence of low-frequency somatic alleles and admixture on caller performance remains understudied. In this paper we therefore aim to evaluate the influence of tumor impurity and heterogeneity on the sensitivity and precision of somatic callers in both whole exome and targeted gene panel data. Because a reference tumor data set with extensively curated low-frequency mutations was not available at the time of this study, we relied on simulated data to be able to distinguish true SNV calls from false ones (false positives) and to evaluate how many mutations were missed (false negatives). Nevertheless, we used clinically relevant cancer mutations to produce realistic tumor data, as detailed below.

Our results provide novel benchmarking data sets that can be used for future testing exercises of heterogeneous and admixed tumor data and represent best practice guidelines to deal with the challenges encountered in clinical tumor samples, particularly those posed by low somatic variant frequencies.

## Materials and methods

### Reference genomes and variant implantation

**Fig 1:**
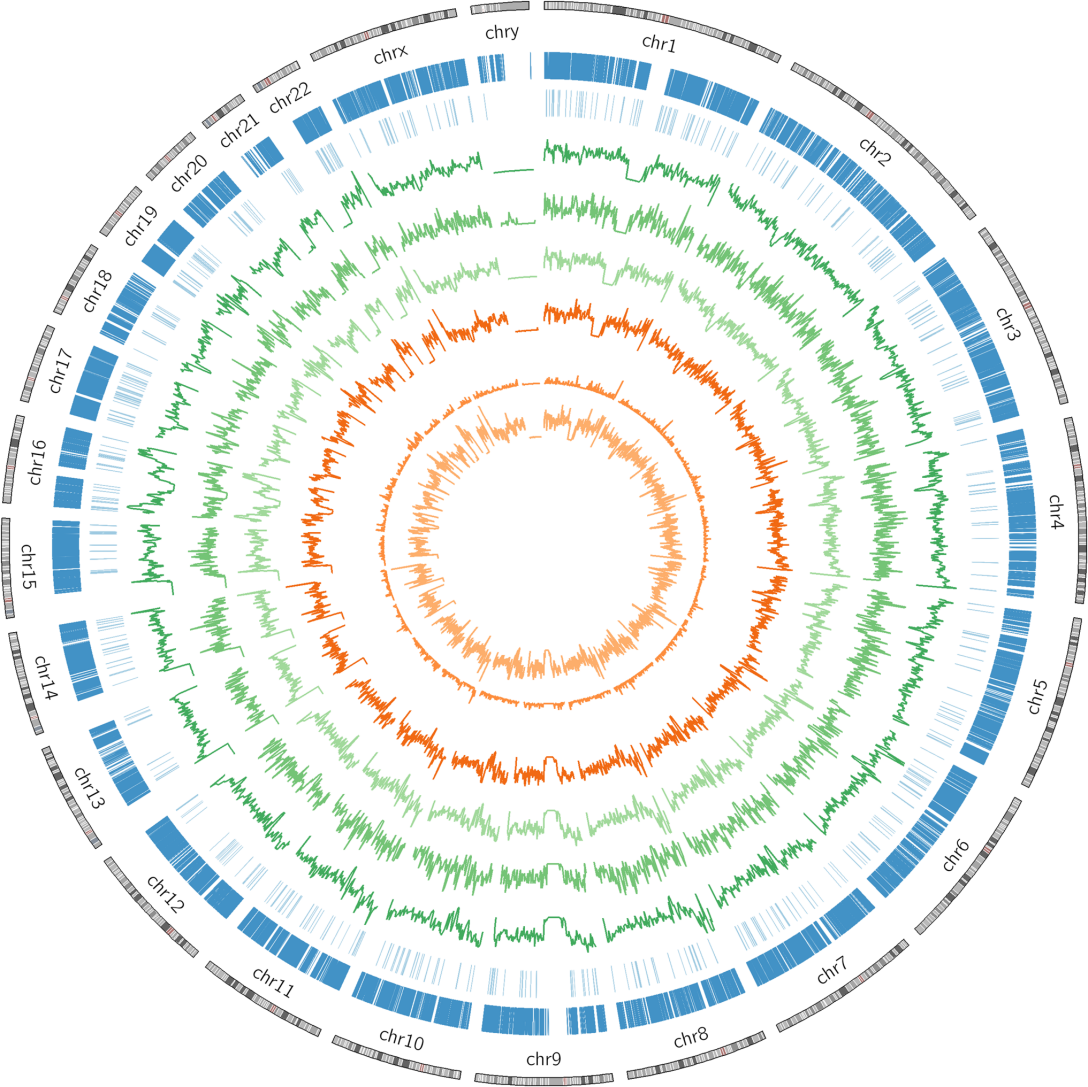
Schematic overview of “gold standard” variants in the simulated data set. Moving from outer to inner circle, the circles show chromosomes, genomic regions covered in the exome experiments (dark blue), genomic regions in the panel regions (light blue), density of germline and somatic SNVs combined (dark green; maximum of scale at 3,000), density of somatic SNVs (green; maximum at 30), density of germline SNVs (light green; maximum at 3,000), density of germline and somatic indels (dark orange; maximum at 300), density of somatic indels (orange; maximum at 30), and density of germline indels (light orange; maximum at 300). Variant densities were computed in 1 Mb bins.

To emulate realistic genomes of cancer patients, we implanted 5,381,311 SNVs, insertions and deletions representing the European-Caucasian ancestry [11, 21] into the GRCh37 human reference genome [22]. The diploid control genome consisted of two independently created genomes representing the maternal and paternal lineage. Tumor data was generated from the control genome by inserting 32,422 known cancer variants from the ICGC and COSMIC [23] databases into our control genome. We repeated this ten times to create five maternal and five paternal cancer genomes, each containing a random subset of the selected cancer variants. To obtain heterogeneous tumor genomes with different allele compositions and frequencies, we combined the five maternal and paternal cancer genomes in randomized proportions. Finally, we simulated ten tumors using a range of 0% to 90% admixture with control sequence. Fig 1 summarizes the variants present in the parental genomes.

### Simulation approach for synthetic genomes

For each tumor data set, we used Wessim [24] to generate 100 bp Illumina reads with a mean insert size of 300 bp (standard deviation 100) for exome and 200 bp (s.d. 50) for panel data, and median coverage of 70x and 520x for exon and panel data, respectively. Wessim applies a sequencing error model with distinct error rates for substitutions, insertions, and deletions, and employs BLAT [25] to mimic probe hybridization. We used Agilent SureSelect Human All Exon v5 probes for whole exome and the Molecular Health Pan-Cancer Gene Panel covering 542 cancer-relevant genes (S1 File) for the gene panel read simulations, which covered 50,390,601 and 2,383,840 nucleotides, respectively. Because the gene panel only covered a limited number of nucleotides, we risked having too few somatic variants for a reliable and sensitive analysis of false negatives. To solve this issue, we simulated a hypermutated tumor for the panel analyses with a high number of somatic variants. After producing the reads, we aligned each data set to the GRCh37 reference using Novoalign 3.04.06 [26] without soft clipping. As recommended by the GATK Best Practices, SNVs were directly called from these alignments using HaplotypeCaller and MuTect2. For all other callers, the alignments were first realigned around indels using GATK IndelRealigner 3.6 [27].

### Choice of SNV callers

We specifically included callers that could deal with paired tumor-control data and that could account for tumor heterogeneity. Moreover, we considered software that is actively maintained and that could be easily installed and run without errors. Our test considered SNV calling methodologies ranging from Bayesian models and heuristic models to statistical tests on variant counts. Using ten simulated tumor admixture data sets and the control, we performed three sets of benchmarking experiments: germline SNV calling from a single (non-tumor) sample, calling any SNVs (germline and somatic) from a single tumor sample without control sample, and somatic SNV calling from paired tumor-control samples. We thus evaluated 11 germline, 10 tumor-only, and 13 paired tumor-control SNV callers. Table 1 provides an overview of the SNV calling tools used in this study. For each tool we tested different settings with respect to minimum coverage, minimum base qualities, minimum mapping qualities, score cutoffs (e.g., p-value), and tool-specific parameters (e.g., model tuning, ploidy). In total, we thus performed 92 germline, 850 tumor-only, and 900 paired tumor-control calling experiments for the exome and panel data set each. The parameters used for each tool are listed in S2 File.

**Table 1:** SNV callers benchmarked in this study. The callers are labelled as the type of lineage they call. Germline denotes calling germline SNVs from a single (non-tumor) sample, tumor denotes calling any SNVs (germline and somatic) from a single tumor sample, and somatic denotes calling somatic SNVs from paired control-tumor samples. *GATK HaplotypeCaller, **GATK Unified Genotyper, ***JointSNVMix outputs both germline and somatic calls from paired control-tumor samples.

Tool	Methodology	Type
Atlas2 1.4.3 [28, 29]	Logistic regression model, platform-specific sequencing and mapping errors	Germline, tumor
deepSNV-1.18.0 [30]	Beta-binomial model	Somatic
EBCall 20160405 [31]	Empirical Bayesian model	Somatic
FreeBayes 20160623 [32]	Bayesian model with error probabilities	Germline, tumor
GATK HC*3.6 [27, 33]	Bayesian model	Germline, tumor
GATK UG**3.6 [27, 33]	Bayesian model	Germline, tumor
JointSNVMix 0.7.5*** [34]	Probabilistic graphical models	Germline, somatic
LoFreqStar 2.1.2 [35]	Statistical test on Poisson-binomial distributed variant counts with error probabilities	Germline, tumor, somatic
MuTect 2015.1-3 [36]	Bayesian classifiers	Somatic
MuTect2 3.6 [36]	Bayesian classifiers	Somatic
QuadGT 20130222 [37]	Bayesian model	Somatic
SAMtools 1.3.1 [38]	Bayesian model	Germline, tumor
Shimmer 20150220 [39]	Statistical hypothesis testing with multiple testing correction	Somatic
SNVMix2 0.12.2 [40]	Probabilistic binomial mixture model	Germline, tumor
SNVSniffer 2.0.4 [41]	Bayesian model	Germline, tumor, somatic
SomaticSniper 1.0.5.0 [42]	Statistical test of genotype likelihood model	Somatic
Strelka 1.0.15 [43]	Bayesan model of admixture	Somatic
VarDict 1.4.6 [44]	Combined heuristic and statistical algorithm	Germline, tumor, somatic
VarScan2 2.4.2 [45]	Combined heuristic and statistical algorithm	Germline, tumor, somatic

### Validation on real sequencing data

Next, we investigated how the SNV callers that had the best results on the synthetic data performed on realistic laboratory data. To be able to calculate sensitivity and precision, we obtained two genomic data sets, HG001 and HG002, for which highly reliable golden standard SNVs are available. The Genome in a Bottle consortium made these high-quality data sets (consisting of reference DNA and validated SNV calls) publicly available specifically for the purpose of analytical validation [46]. To produce admixed data, DNA from the two samples were mixed at the ratio of 1:7; this was repeated for four replicates. Agilent capture libraries were prepared for the Molecular Health Cancer Gene Panel and sequenced on an Illumina HiSeq 2500 machine with 101 bp paired-end reads and average insert size of 166 bp. The reads were aligned to the GRCh37 reference using Novoalign 3.04.06 [26] without soft clipping, duplicates were removed using Picard MarkDuplicates 2.5.0 [47], and realigned using GATK IndelRealigner 3.6 [27]. The median coverage was around 1,400x. Finally, SNVs were called using LoFreqStar 2.1.2 [35], VarDict 1.4.6 [44] and VarScan 2.4.2 [45], which were the three best SNV callers in terms of sensitivity and precision on the synthetic panel data sets. To calculate true and false positives, we compared the SNV calls to the GiaB gold standard data set. The latter consisted of the union of the high-confidence gold standard SNVs for HG001 and HG002 that fall within the genomic areas covered by the Molecular Health target regions. In total, the data set contained 1,363 gold standard SNVs.

### Evaluation measures

For each caller and data set we compared the SNVs obtained from the caller with the known “gold standard” variants we implanted into the data sets. For the germline and somatic callers we compared against the known germline and somatic SNVs, respectively. For the tumor callers, we restricted the evaluation on somatic SNVs as the performance of calling germline SNVs, which are more abundant compared to somatic SNVs, would mask the performance of calling somatic SNVs. We determined the number of (i) true positives (TP), or SNVs called at the correct position; (ii) false positives (FP), or SNVs called but not present in the gold standard set and (iii) false negatives (FN), SNVs that were not called by the evaluated method. Using these counts, we calculated (i) sensitivity, $\frac{TP}{TP+FN}$, the ability of a caller to find true SNVs and (ii) precision, $\frac{TP}{TP+FP}$, the proportion of true SNVs relative to all SNVs found by the caller. We determined precision and sensitivity of each caller for different data sets (admixture levels) and tool settings. For each dataset, each tool and each set of parameters we calculated the harmonic mean of the sensitivity and precision as a measure of parameter performance. In the manuscript, we only report the highest (best-performing) parameter settings for each tool, data set and, where appropriate, admixture level; the full results can be found in the supplement.

## Results

### Alignment and data set properties

**Fig 2:**
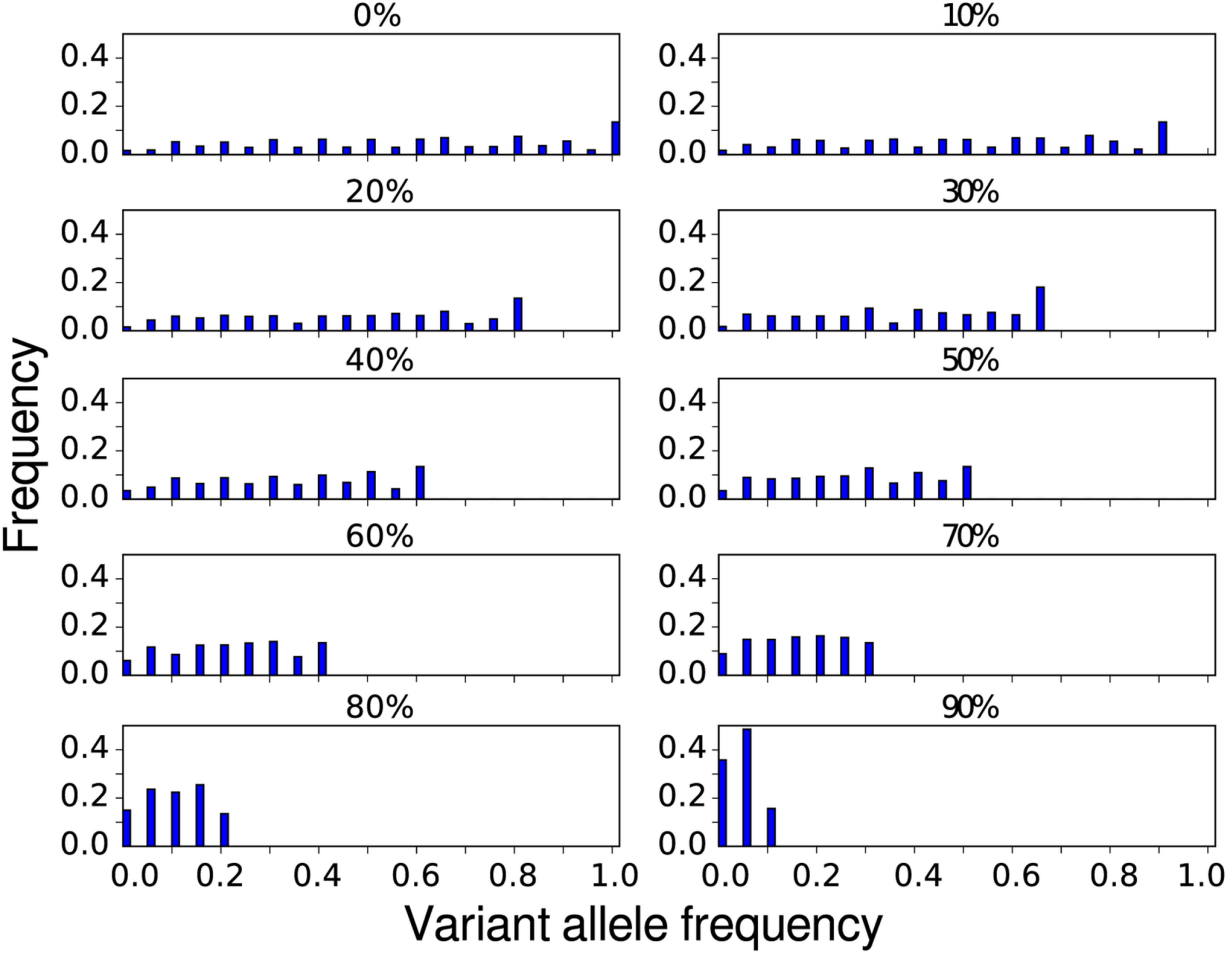
Histograms of true allele frequencies in each tumor sample. Note how increasing admixture increases the prevalence of low-frequency variants.

We obtained one control and ten tumor data sets with admixture levels varying between 0 (pure tumor) and 90% (mostly contamination with normal tissue). Fig 2 shows the range of variant frequencies present in each of the data sets. The final data sets used as control samples contained 59,664 and 2,425 germline SNVs for exome and gene panel, respectively. For the tumor samples these numbers were 636 and 3,497 SNVs, respectively. The latter number is so high because we had to ensure a reliable analysis of false negatives in the panel data (see Methods).

### SNV caller benchmarking of germline data

For each caller, we compared a set of 1 to 13 parameter settings, depending on the number of available parameters (S2 File). In the main text and figures we will only discuss the optimal (best-performing) parameter set for each individual caller. The optimal parameter set had the highest harmonic mean of the sensitivity and precision. For a complete overview of results across tools and parameters, we refer to the Supplement S3 File.

**Fig 3:**
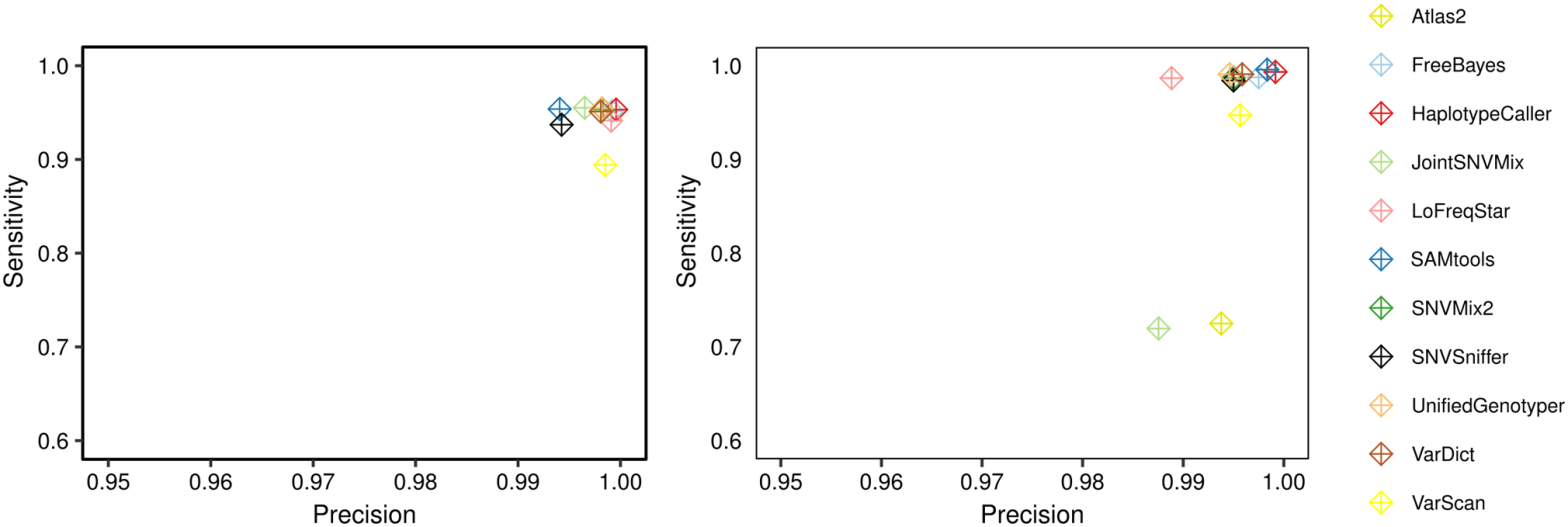
Benchmarking results for germline SNVs. Sensitivity versus precision is shown for A. exome and B. targeted gene panel data.

For germline data, all but three callers had sensitivity and precision above 90% and 99% on exome data, respectively, and even higher average sensitivities on panel data for similar precision (98%, 99%; Fig 3A). Evaluation results for Atlas2 on exome data are missing as the experiments were not finished successfully. The worst performance on exome data was seen for VarScan2 (89.3% sensitivity). The best callers for exome data were HaplotypeCaller, which correctly called 56,869 out of 59,664 gold standard mutations with 24 false positives (sensitivity 95.3%, precision 100.0%), and FreeBayes with 56,775 correct and 47 false SNV calls (95.1%, 99.9%). Interestingly, this means that the best methods still missed more than 2,500 mutations that were present in the simulated germline exome data.

On high coverage targeted gene panel data (Fig 3B) the best performance was seen for HaplotypeCaller (99.3% sensitivity and 99.9% precision), SAMtools (99.6% sensitivity and 99.8% precision), and FreeBayes (98.8% sensitivity and 99.8% precision). Atlas2 (72.5% sensitivity), and JointSNVMix (72.0% sensitivity) identified the lowest number of SNVs.

Next, we looked at the concordance of calls between all callers. For the exome data, all ten germline callers correctly identified a core set representing 86.6% of SNVs without false positives (S1A and S1B Fig). On the panel data, 44.6% of SNVs were identified by all eleven callers, and 91.6% by at least ten callers at very high precision (> 99.9%).

### Influence of admixture on tumor SNV calling

We also systematically compared up to 13 distinct parameter combinations for each tool and each somatic data set (S2 File). For the sake of brevity and to avoid confusion, we here present the best performing parameter set for each tool at each admixture level. Again these were the settings with the highest harmonic mean of sensitivity and precision for a particular tool at a particular level of admixture. Therefore it is possible that sensitivity and precision values calculated at different admixtures were reached by tweaking the appropriate parameters. The complete results can be found in the Supplement S3 File.

**Fig 4:**
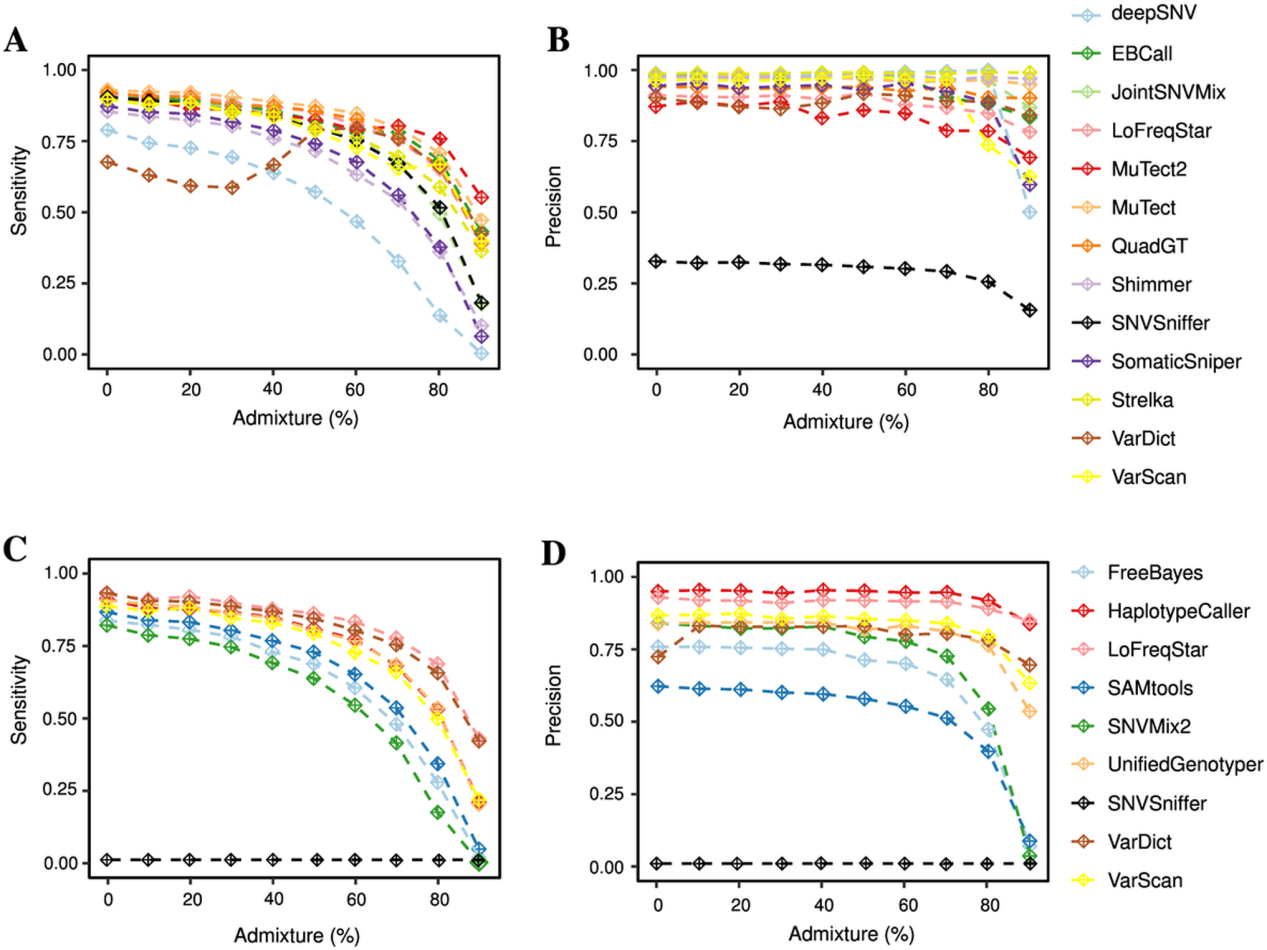
Benchmarking results for somatic SNVs on exome data. A and C. Sensitivity; B and D. precision for somatic SNVs. A, B. on paired tumor-control exome data; C, D. on single tumor exome data.

Compared to germline calling, the low frequencies of many alleles in the paired tumor-control whole exome data set led to significantly lower sensitivity and precision for all tools (Fig 4A and 4B). About 80.7% of known SNVs were correctly identified by all 13 exome callers or all except one (S1C Fig) in the pure tumor data. Only six out of 13 tools managed a sensitivity above 90% (Fig 4A); of these, EBCall, JointSNVMix, MuTect, LoFreqStar and QuadGT were able to also reach more than 90% precision (Fig 4B). Given 636 gold standard SNVs, e. g., MuTect called 592 with 14 false positives, and LoFreqStar 582 with 56 false positives. VarDict performed worst, missing 206 mutations and falsely calling 46.

Increasing levels of tumor admixture resulted in rapily declining sensitivity for all tools. At intermediate (50%) admixture, all tools together were able to find 90.1% of SNVs (S1C Fig). At 90% admixture, the seven best callers called between 36.3% and 55.2% of the SNVs, and all tools together called 58.0%. This effect was less pronounced for precision, with almost negligible differences in precision for Strelka, deepSNV, Shimmer and MuTect at different admixture levels. In all, MuTect showed the best overall performance for paired tumor-control exome data at all admixture levels, closely followed by Strelka and QuadGT. This illustrates that most tools had trouble identifying low-frequency variants in admixed tumor exome data.

We next looked at the performance of SNV calling on a single tumor exome sample. Although these tools called both somatic and germline SNVs, we focused the sensitivity and precision analysis on the somatic calls only. Here, we also saw declining sensitivity with increasing admixture levels for all tools (Fig 4C). The effect on precision was less pronounced for about half of the tools; for the other half precision started falling at around 60% contamination with normal tissue (Fig 4D). HaplotypeCaller and LoFreqStar maintained the highest precision across all but the lowest admixture levels. Overall, LoFreqStar showed the best performance in terms of sensitivity and precision.

**Fig 5:**
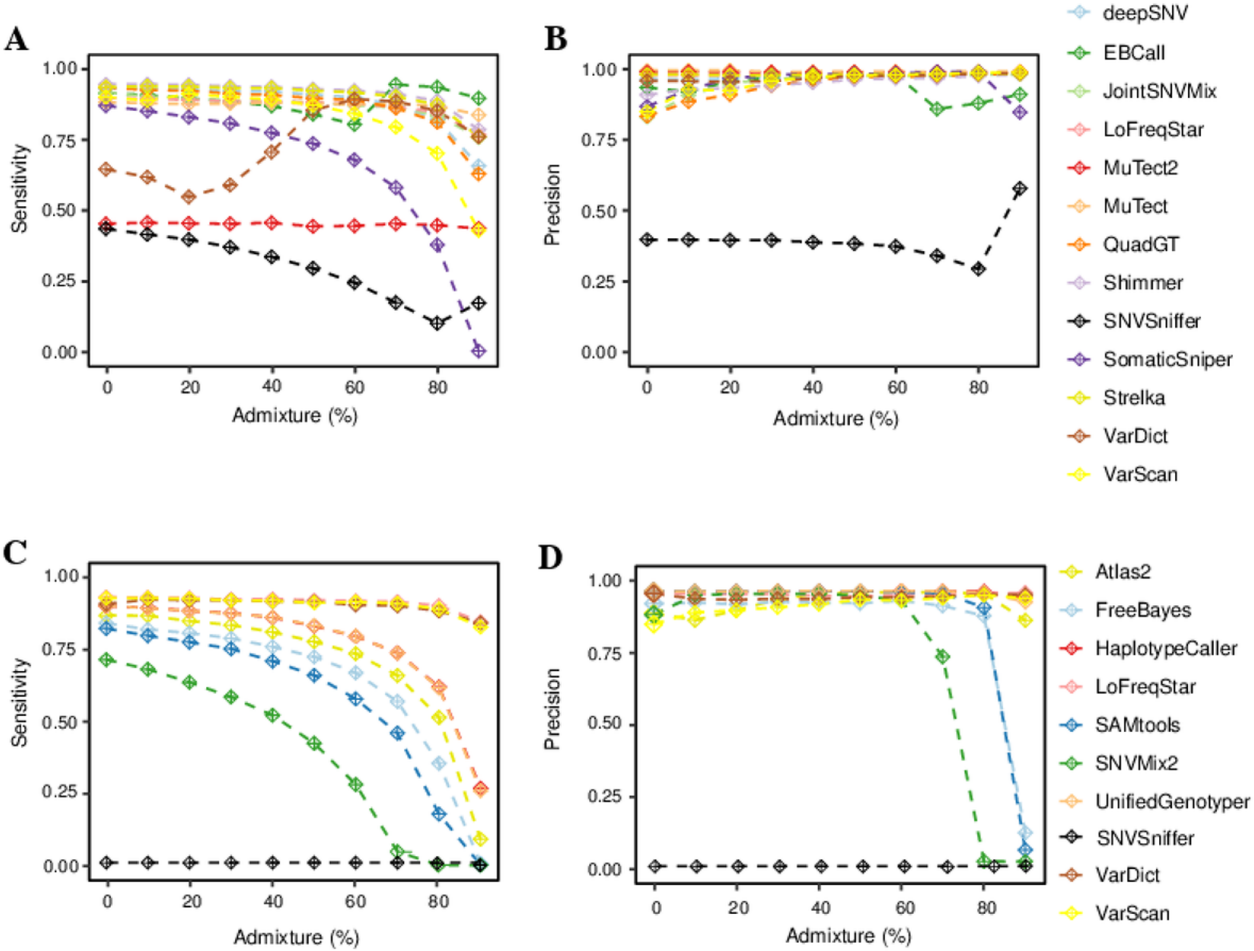
Benchmarking results for somatic SNVs on targeted gene panel data. A and C. Sensitivity; B and D. precision for somatic SNVs. A, B. on paired tumor-control targeted gene panel data; C, D. on single tumor targeted gene panel data.

Although this is rarely done in clinical practice, we also investigated the sensitivity and precision of calling SNVs from paired tumor-control targeted gene panels. In these experiments, we observed an increased senstivity compared to the exome data, particularly for increasing levels of contamination of normal tissue (Fig 5A). Interestingly, MuTect2 and SNVSniffer were much less sensitive on paired panel data than on exome data. Moreover, precision was above 90% for almost all callers and admixture levels (Fig 5B). EBCall reached high sensitivity in highly admixed tumors when it was provided with an estimate of tumor allele frequency. However, it remains unclear whether tumor cellularity estimates based on pathological samples would yield similar improvements on realistic data.

Finally, we investigated the performance of SNV calling on more typical (unpaired) targeted gene panel data, which excludes a control sample but has increased coverage. With this type of data we also saw high variability among callers and declining sensitivity with increasing admixture levels for all tools (Fig 5C and 5D). Precision was very high for all tools and admixture levels, except for SNVSniffer, SNVMix2, SAMtools and FreeBayers for admixture levels above 60%.

### Impact of parameter settings on detection of low-frequency somatic SNVs

Our comparison of parameter settings across tools and data types showed that parameters related to base-quality cutoffs can be used to tweak the balance between finding low-frequency SNVs and avoiding false positives. Too high cutoffs resulted in loss of low-frequency SNVs, whereas too low ones introduced false positives. Nevertheless, in germline exome data, base quality thresholds only slightly lowered the sensitivity of HaplotypeCaller and SAMtools. In all of the somatic SNV experiments, raising base quality and variant coverage thresholds increased sensitivity to somatic variants at the cost of additional false positives. At the highest admixture levels (starting from 70%), the influence of parameters was negligible because the calls were dominated by false positives.

### Validation of the three best performing algorithms using GiaB reference DNA and gold standard SNVs

**Fig 6:**
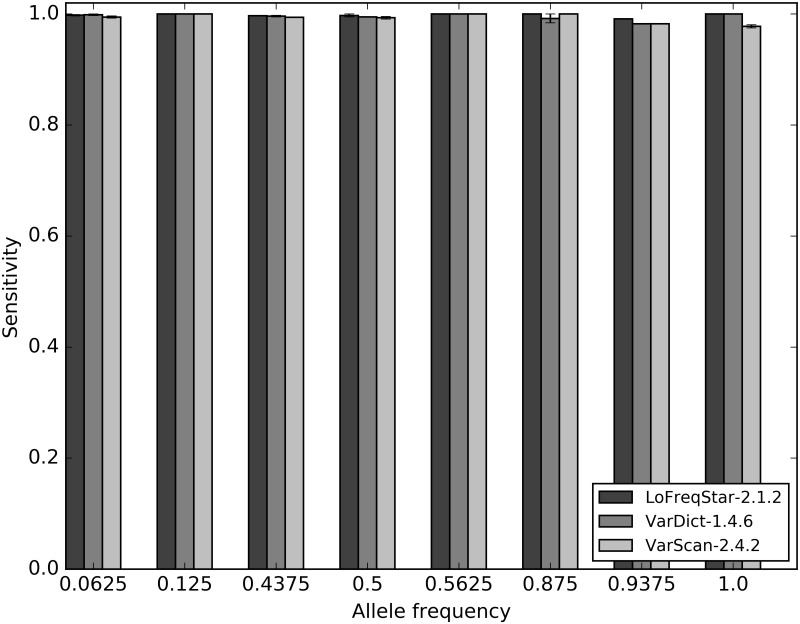
Sensitivity of LoFreqStar, VarDict and VarScan on the GiaB reference samples averaged over the four replicates. Because of the 1:7 mixtures, allele frequencies are discrete for the given values.

The GiaB consortium provides reference DNA with associated validated SNV calls that can be used as golden standard for evaluating novel analytical approaches against a known standard. We tested LoFreqStar, VarDict and VarScan, which performed best on targeted panel sequencing data derived from the synthetic genomes, on sequences derived from 4 replicates of 1:7 proportional mixtures of the DNA of the two GiaB reference samples (Table 2, Fig 6). On average, LoFreqStar again performed best, and identified 1,501 SNVs, which resulted in an average sensitivity of 0.998 and an average precision of 0.899, which is slightly lower than on the synthetic data sets. VarDict and VarScan performed similarly on GiaB as on synthetic data. Just as was the case for the synthetic targeted panel data, the sensitivity was not strongly influenced by the allele frequencies in the data set. These results confirm that LoFreqStar is a SNV caller that performs well on targeted gene panel data, even if the samples are admixed and most variants have low frequencies.

**Table 2:** Sensitivity and precision of LoFreqStar, VarDict and VarScan on GiaB reference samples. The SNVs predicted by the algorithm were compared to the golden standard SNVs provided by GiaB.

Tool	Replicate	SNVs called	Sensitivity	Precision
LoFreqStar	1	1,483	0.997	0.909
	2	1,497	0.998	0.901
	3	1,516	0.998	0.890
	4	1,509	0.998	0.894
VarDict	1	1,512	0.996	0.890
	2	1,513	0.996	0.890
	3	1,525	0.996	0.883
	4	1,510	0.996	0.892
VarScan	1	1,480	0.992	0.906
	2	1,501	0.993	0.894
	3	1,492	0.992	0.899
	4	1,488	0.993	0.902

## Discussion

Genomic pipelines in cancer precision medicine need to discover all cancer-relevant mutations while avoiding misleading false positives. This study aimed to investigate the impact of low somatic allele frequencies and tumor-germline admixture on the sensitivity and precision of somatic SNV calling. Based on a comprehensive and systematic analysis of 19 state-of-the-art tools each with up to 13 distinct parameter settings, we here present a set of best practice recommendations for optimizing somatic SNV calling from tumor samples.

We found vast differences among tools regarding the number and type of calls across germline and somatic SNVs, both in whole exome and in targeted panel data. Previous studies also reported that somatic callers tend to identify a small, common set of high-confidence core mutations [16, 18] combined with an idiosyncratic set of either false positives (VarScan2, SomaticSniper) or low-frequency true positive mutations (MuTect, Strelka) [19]. A popular caller, VarScan2, has difficulties with detecting lower-frequency mutations [16, 17, 19], often calls germline mutations [16] and is systematically outperformed by other tools on both whole exome and gene panel data. Taken together, our results emphasize that identification of cancer-relevant somatic variants requires methods that are specifically tailored to a particular type of experiment (whole exome, targeted gene panel) using parameters that avoid calling too many false positives (minimum variant coverage, base quality thresholds, expected tumor heterogeneity, admixture; see below).

Based on our analysis of about 3,700 SNV calling experiments and the literature, we therefore recommend the following best practices. For whole exome germline data, HaplotypeCaller and FreeBayes are the most reliable tools. On germline gene panel data, SAMtools is slightly more sensitive than the other two. For tumor whole exome data, joint tumor-normal calling using MuTect optimizes sensitivity and minimizes false positives. However, MuTect does not report germline variants, which could be hereditary cancer-relevant mutations such as BRCA1, BRCA2, TP53 or HER2 [48–50]. Failure to identify such germline cancer mutations may lead to imprecise treatment recommendations. MuTect should therefore be combined with a HaplotyperCaller analysis of the normal (germline) sample. Also Strelka, EBCall and QuadGT are reliable, but slightly less sensitive alternatives to MuTect for whole exome analyses. Targeted gene panel data exhibit higher coverage than exome data and should thus be used to improve detection of low-frequency variants. However, this comes at the cost of many missed variants in regions not captured by the panel and less straightforward detection of germline mutations. On this type of data, about half of the tested tools showed very good performance, both on synthetic and realistic lab-derived and admixed GiaB data. For single tumor panel data we recommend using LoFreqStar or VarDict, with appropriate base quality and variant coverage thresholds, especially considering that in clinical practice targeted panel sequencing does not include control samples.

The analysis of parameter settings across tools further demonstrated that the quality and purity of the sample had a much stronger influence on the performance of the SNV callers than particular settings. Similarly, algorithm choice had more impact on the discovery of low-frequency variants than tweaking parameters such as minimal variant coverage and base quality scores in any particular tool. Nevertheless, adjusting these parameters for the tool that is optimal for a given type of data does allow the researcher to balance sensitivity and precision according to his or her preferences, but this only impacts a very small (but potentially informative!) fraction of the total variants called by any method.

Combining caller outputs for somatic variant calling did not improve results much, as the most sensitive callers called both “core” and low-frequency mutations, whereas other tools produced many false positives. This remains a contentious issue, with several authors agreeing in favor [16, 17, 20] and some against caller integration [18]. The choice for either may be strongly influenced by the type of caller (LoFreq and MuTect were not included in all studies, for example) and the type of data that was tested, with confidence in combining callers often recommended for well-curated data sets with little admixture.

Our results emphasize that precision medicine pipelines need to pay special attention to admixture of tumor samples with germline cells, especially for exome data with low coverage. Admixture may be caused by biological factors, as tumor tissues often contain epithelial, stromal and vascular cells that play a role in tumor growth and progression [51]. Moreover, immune cells may penetrate tumor tissue to exert tumor suppression or may be coopted by the tumor for tissue invasion and metastasis [52, 53]. On the other hand, admixture may be an artifact of pathological biopsies, which can exhibit 60% to 90% germline contamination [54] (tumor cells thus sometimes constitute a minority of the sample). This has two important consequences for optimization of genomic pipelines. First, high fractions of germline variants complicate separation of somatic and germline variants, which is particularly problematic if the germline includes cancer-predisposing [48, 55] or resistance mutations [56]. This is most easily mitigated by joint tumor-control sampling [55]. Second, admixture reduces somatic allele frequencies: a somatic variant present at a true frequency of 30% in the tumor only has a 3% allele frequency in a highly contaminated biopsy and still only 13.3% in a “good” sample. Our analysis demonstrates that admixture can severely impact sensitivity, and may negatively influence precision in exome sequencing projects. Regardless of the tool used, admixture leads to a reduction in overall calls, and an exponential drop in the sensitivity for calling somatic mutations. With 70% admixture, the best caller (MuTect) misses over 14% of mutations, even if the variants were initially present at 100% of the tumor. At high levels of admixture, high-coverage targeted panels may thus recover more variants than whole exome approaches. Each patient sample thus benefits from contextualization of the analysis pipeline, such that the appropriate balance can be struck between recovering low-frequency variants (gene panels) and avoiding germline variants (paired tumor-normal whole exome data).

Taken together, our results caution that admixture represents a significant quality issue for precision medicine that may hamper the ability of pipelines to deliver complete, reliable and actionable results. Unfortunately, current clinical practice often does not foresee later genetic analysis [54]. The future of precision medicine thus hinges as much on increased quality and purity of tumor sampling as it does on optimized and sensitive analyses. Genomic pipelines should therefor include a tumor purity check to accommodate large variations in somatic allele frequencies and germline contaminations.

Finally, low frequency variants may also be an intrinsic property of genetically heterogeneous tumors [12–14]. At the moment of clinical diagnosis, tumors consist of spatially distinct subclones that evolved from the most recent ancestor through mutation, selection and adaptation to the changing environment around the growing tumor. Although low-frequency variants in subclones are very difficult to detect, their presence in the tumor is highly relevant for tumor progression, metastasis and thus therapy [57–60]. Cancer treatment effectively suppresses the drug-sensitive dominant clone, leaving only the few drug-resistant cells that typically had a growth disadvantage compared to the sensitive cells dominating the primary tumor [61–63]. The removal of competition from fitter, drug-sensitive cells subsequently enables uninhibited growth of an often more aggressive, drug-resistant secondary tumor [62, 64, 65]. Therefore cancer treatments often only buy a few months of progression-free and limited overall survival. A recent comprehensive study of hundreds of patients across cancer indications confirmed a highly significant relationship between intra-tumor heterogeneity and mortality [66]. The identification of rare subclones carrying resistance biomarkers is thus of high clinical relevance, and may be exploited to favor therapies that mitigate the evolutionary advantage of resistant cells during treatment [67, 68]. Tuning of clinical genomic pipelines to reliably detect low-frequency SNVs in heterogeneous tumors may thus be crucial to optimize long-term treatment success of heterogeneous tumors. Since our results show that most of the tested algorithms could not reliably detect low-frequency variants in exome data, but were much more sensitive on the increased coverage of targeted panel data, the analysis of highly heterogeneous tumors may benefit from a sensitive, targeted sequencing design with increased coverage.

## Conclusion

In conclusion, our study of SNV callers within the context of low-frequency somatic variants showed that developing reliable genomic pipelines is far from trivial. Whole exome analyses offer the advantage of covering a large part of the genome, which increases the likelihood of finding (rare) cancer-relevant mutations. It often includes a non-cancerous tissue or blood sample that allows efficient separation of somatic from germline mutations and the determination of cancer-predisposing germline mutations. Targeted gene panels are more cost-effective than whole exome data and are more sensitive to low-frequency variants due to higher coverage, but do not always allow efficient identification of germline variants and by definition miss somatic variants in genomic regions not covered by the panel. Based on a case-per-case basis, genomic pipelines need to balance a tradeoff between sensitivity to low-frequency variants and calling too many false positives, according to the type, quality and admixture of the data that is being analyzed. Finally, we caution that the reliability of precision medicine pipelines depends at least as much on the quality and purity of the tumor sample as on optimization of the methods and parameters within the pipeline. Using a standard pipeline that is not optimized for the data at hand as a first step in precision medicine risks missing relevant cancer mutations and may negatively impact the quality of treatment recommendations.

## Supporting information

S1 Fig Concordance among SNV callers. Concordance among SNVs callers for exome and targeted gene panel data. A. Germline exome (left) and germline targeted gene panel (right). B. Paired tumor-control exome, C. single tumor targeted gene panel, D. paired tumor-control targeted gene panel data, and E. single tumor targeted gene panel. Each slice represents the proportion of calls shared by the corresponding number of callers relative to the total calls made by all callers. The legend gives the number of callers supporting a set of calls. Numbers above pie charts represent different admixture levels (0 to 90%).
(TIFF)

S1 File Panel gene list. Molecular Health Pan-Cancer Gene Panel covering 542 cancer-relevant genes.
(TXT)

S2 File Tools parameters. Contains the parameters used for running the SNV callers.
(ZIP)

S3 File Evaluation. Contains the evaluation of SNV calls per caller and parameter set combination.
(ZIP)
