# Supplementary figures and images for: Comprehensive benchmarking of SNV callers for highly admixed tumor data
Source: PLoS One. 2017 Oct 11;12(10):e0186175. doi: 10.1371/journal.pone.0186175 (PMC5636151; doi:10.1371/journal.pone.0186175)

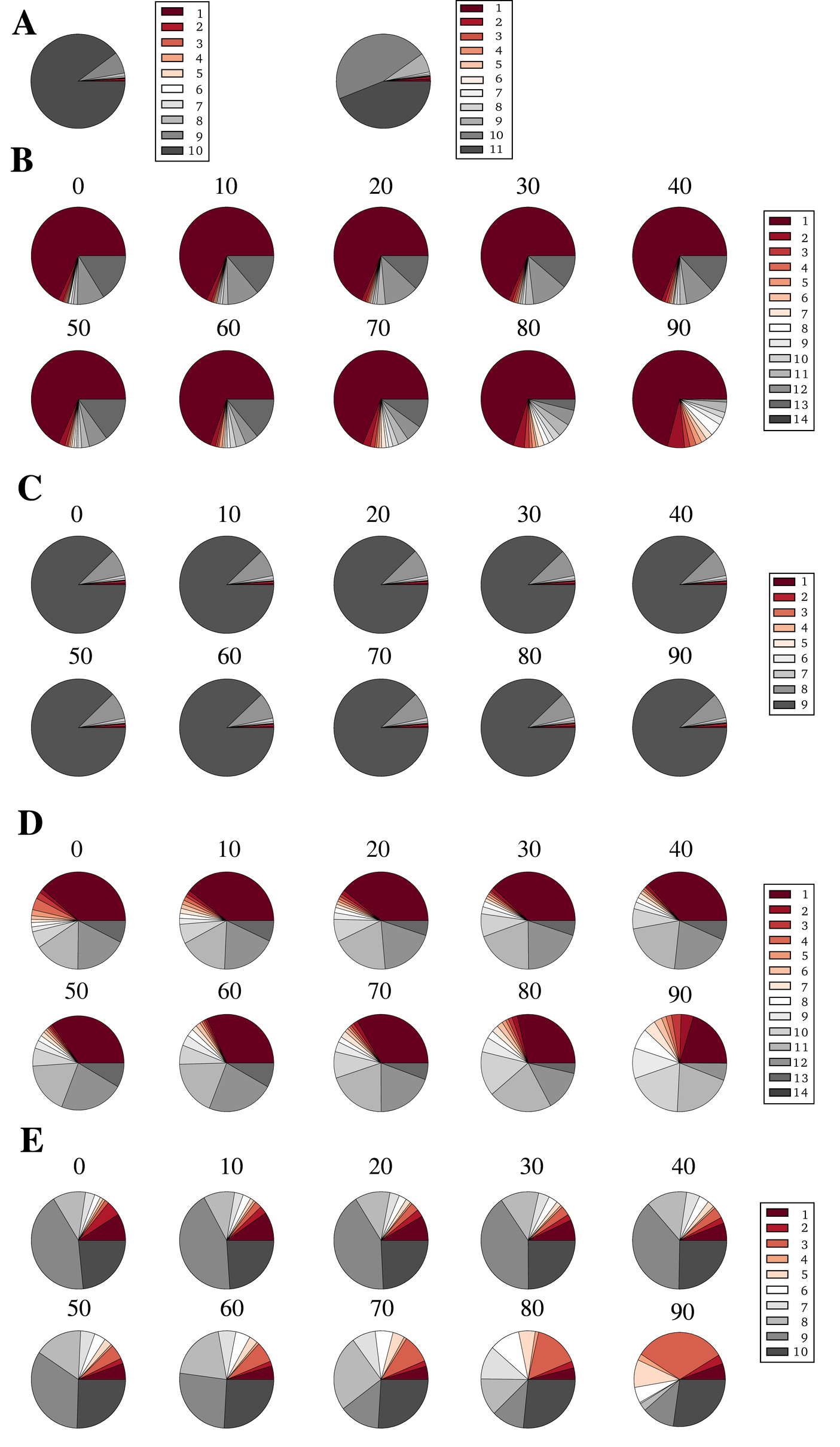

Supplement: S1 Fig — Concordance among SNVs callers for exome and targeted gene panel data. A. Germline exome (left) and germline targeted gene panel (right). B. Paired tumor-control exome, C. single tumor targeted gene panel, D. paired tumor-control targeted gene panel data, and E. single tumor targeted gene panel. Each slice represents the proportion of calls shared by the corresponding number of callers relative to the total calls made by all callers. The legend gives the number of callers supporting a set of calls. Numbers above pie charts represent different admixture levels (0 to 90%). (TIFF) [file pone.0186175.s001.tiff]
